# 

**Published:** 1888-01

**Authors:** 


### Society Notes.

### TEXAS STATE MEDICAL ASSOCIATION.

[The following address to the profession of Texas, by the President of the Texas State Medical Association, has been issued from the Secretary's office in the form of a circular letter: Ed.]

Executive Office, Raymond, Texas, January 20, 1888.

Dear Doctor:—It is a pleasure, as well as my duty, to announce that the Twentieth Annual Session of the Texas State Medical Association will be held in the city of Galveston, beginning on the fourth Tuesday in April, 1888.

Galveston is the chief and most important seaport city on the coast of Texas, and in addition to its other numerous attractions, all of which are prone to please and interest the observer, it is peopled with a citizenship rich in intelligence and culture, rich in charity and unmeasured hospitality, and rich in enterprise and means. It possesses unsurpassed facilities for the handsome entertainment of large delegations, and our able Committee of Arrangements will spare no pains in making everyone present happy, and realize "that it is good for him to be there;" then, meet us at the seaboard, and induce your medical friends to come with you, to the end that our strength may be increased, and our efficiency perpetuated.

Aside from the anticipated social and scientific feast that will doubtless await all who may attend, there are other considerations which should prompt the attendance of every member, challenge and command his best thought.

Chief among these will be the consideration of the Report of the Committee on Revision of Constitution and By-Laws. Some radical changes are proposed in the Report, and you are most earnestly requested to study them comparatively, and in all their relations, and come prepared to contribute your counsel and cast your vote.

To maintain in perpetuity the high plane of recognition, distinction and usefu,ness, to which the Texas State Medical Association has been elevated by your zeal and concentrated efforts, will require incessant vigilance and labor on the part of the membership.

Distinguished members of our profession from other States have been solicited to be in attendance, to swell our number, augment our joys, and add lustre to the social and scientific entertainmants of the occasion.

Therefore, in the interest of your State organization, scientific medicine in general, and that harmony which should pervade the ranks of our vocation everywhere, come! From the beautiful fruit and grain growing regions of the North; from the extensive and fast developing plains of the attractive West; from the broad and fertile pampas of the balmy South, and from the flowers and foliage of a Texan Orient, and give us the "light of your countenance," a cordial grasp of the hand, the pleasure of your society, and aid of your counsel. I have the honor to remain

Your most obedient servant,

SAM. R. BURROUGHS, M. D.,
President Tex. State Med. Association.

